# Gender Differences in Non-sex Linked Disorders: Insights From Huntington's Disease

**DOI:** 10.3389/fneur.2020.00571

**Published:** 2020-07-07

**Authors:** Daniel Zielonka, Barbara Stawinska-Witoszynska

**Affiliations:** ^1^The Department of Public Health, The Poznan University of Medical Sciences, Poznań, Poland; ^2^The Department of Epidemiology and Hygiene, The Poznan University of Medical Sciences, Poznań, Poland

**Keywords:** Huntington's disease, gender differences, sex contribution, autosomal, monogenetic

## Introduction

Gender plays a role in the prevalence and natural course of several disorders. It is apparent in neurodegenerative diseases like Parkinson's disease more prevalent in men, Alzheimer's disease more prevalent in women, or Lewy body dementia more prevalent in men. Rarely, however, recently more often, in autosomally conditioned diseases, gender differences are being identified (1–21).

Huntington's disease (HD) as a rare neurodegenerative (recently reported peripheral tissue involvement), incurable—therefore still displaying natural course—disorder with an autosomal dominant pattern of inheritance with full penetrance in most cases (22). Therefore it was not explored for gender differences for many years. Gender was considered in HD, however, with respect to a parent of an HD patient, as it was observed that disease inherited from a father resulted in symptoms anticipation, namely earlier onset and faster progression than when inherited from the mother (23–25). It was later explained by a higher probability for elongation of the causative HD mutation during spermatogenesis, than during oogenesis (26, 27). Very quickly after identification of a causative mutation in 1993, it was observed that there is a negative correlation between the number of CAG repeats in the causative gene (expansion of causative mutation) and onset age, namely larger expansion of CAG repetitive sequence in the HD gene resulted in earlier HD symptoms onset (28). The rate of HD progression was explored early after the causative gene identification. Interesting results were observed in small groups (29–31), but lack of proper tools for progression measurement resulted in a lack of any results indicating a correlation between CAG repeats expansion and rate of the disease progression (32–35). First important (36) and deciding findings confirming that the rate of HD progression is dependent on CAG repeats number were described in 2008 in a large HD cohort study (37). In the same year, another small study indicated gender differences in HD patients (38).

## Sex Plays a Role

The finding described in the research paper published in 2008 (38) came rather as a surprise during the study's data analysis (38). First, the study was based on just 41 HD patients: 24 women (38). A statistically significant correlation between the number of CAG repeats and scores in Unified Huntington's Rating Scale (UHDRS) subscales—motor, cognitive, functional, independence and total functional capacity (TFC)—were identified in women but not in men. Moreover, time from onset correlated with scores in above listed UHDRS subscales in women only. These findings provided insight allowing further investigation to study gender differences in HD.

In 2013, a large cohort analysis based on 1,267 HD affected individuals was performed (39). This study based on data collected in REGISTRY, an Observational Study of the European Huntington's Disease Network (EHDN) (40) population, and was aimed to identify gender differences in several HD features including differences in the rate of the disease progression based on following annual visits when patients were assessed in UHDRS subscales. The study was controlled for several environmental factors. The most important finding of this study was identification of a significant gender difference in the rate of HD progression controlled among other things for disease burden (calculated variable incorporating CAG repeats number in larger allele and age): DB = (CAG number in the larger allele – 35.5) × age in years (41). Disease burden reflects the stage of brain pathology in HD and includes the factor responsible for 70% of the variability in HD onset, namely CAG repeats number in mutated *HTT* allele (42, 43). Both genders did not differ for the disease burden or for onset age, but the progression rate in women was faster. Other gender differences were identified in cross-sectional analysis for years of education (men studied longer), presence of depression (in women more often), history of depression (in women more often), alcohol abuse (more often in men), and cigarette smoking (more often in men). To explain the observed difference in the rate of HD progression, the longitudinal analysis was controlled for several variables like disease duration, years of education, presence of depression, depressive episodes in the past, history of psychotic disturbances, history of obsessive-compulsive disorders, history of suicidal ideation, smoking, alcohol abuse, and drug usage. These variables were used as confounders in the longitudinal analysis did not clarify the inter-gender differences in progression rate. The above-described study provided solid evidence to confirm the presence of gender differences in HD clinical picture and what is more important in rate of HD progression.

Above mentioned findings were confirmed and explored in more detail in 2018 in another study, which aimed at a gender effect in particular symptomatic domains of HD and their contribution to functional abilities and quality of life (44). The clinical picture of HD is formed by three major symptomatic domains, namely motor symptoms (e.g., clumsiness, chorea, dystonia), cognitive impairment (subcortical dementia), and behavioral disturbances (e.g., depression, apathy, irritability, and aggression) (22). All of these domains contribute to functional abilities of affected individuals but the impact of a particular domain for progression of functional disability was not clearly explained in previously published studies (45–48). Considering the study (39), it was important to evaluate also gender role in this contribution. The study (44) based on 2,191 HD affected individuals (1,080 women), REGISTRY study's participants, annually examined in UHDRS for several years, was controlled for the same factors as previously described study. In women significantly stronger correlations between all symptomatic spheres and HD progression rate was observed, motor domain contributed the most, followed by cognitive and behavioral; moreover, motor symptoms were responsible for more variability in functional abilities in women than in men, while cognitive symptoms had an opposite contribution (more variability in men than in women). This means that motor symptoms were the strongest contributors to functional abilities in both genders, particularly in women, although cognitive symptoms are more important in men for functional abilities than in women.

One year after the previously described research paper was published, another cohort study delivered surprising results (49). Here it was observed in a huge cohort of 67 millions of Americans performed between 2003 and 2016 that HD has a significantly higher prevalence in women estimated on 7.05 per 100,000 than in men, 6.91 per 100,000. This result may suggest a more severe HD pathologic process in women.

There is also evidence based on pre-clinical settings where in animal HD models gender differences were identified. The first important research paper was published in 2007; in the murine model, it was observed that a lower level of extracellular ascorbate in the striatum in males reflected a more severe phenotype than in females (50). In 2008 another study suggested the neuroprotective effect of 17b-estradiol in females in rat HD model (51). In 2016 in BACHD mouse model of Huntington's disease, it was found that circadian activity levels, rhythm precision, and behavioral fragmentation are more severe in males (52). Finally, in 2019 more severe deficits in neuroprotective nitric oxide synthase activity in the HD cortex and striatum were observed mostly in Q175 males of HD mouse model (53). In contrast to human studies, animal research results indicate a more severe picture in males.

## Discussion

Above mentioned studies indicate presence of gender differences in HD. The limitation factor of the finding (39, 44) is a lack of inclusion of two important, however difficult to be assessed variables, namely concomitant disorders and medications. There is still a question whether differences in these two variables could explain gender differences reported so far (39, 44). Depression was considered as a confounder in both studies but did not explain differences in HD progression rate between genders. The prevalence of non-psychiatric concomitant disorders does not differ between genders as reported in recently published paper (54). It seems that both genders are not treated differently in HD but this should be explored in detail.

Also animal studies, confirming gender differences, bring contrary results of gender burden, displaying their ample limitations in explanation of pathologic processes behind observed differences (50–53). The findings in animal models suggest their imperfection rather (55). Hormonal disturbances observed in an animal model (51) and in HD patients (56, 57) could be supportive to explain the phenomenon, but a cause of the gender differences in HD seems to be more complex and require future studies in larger HD cohorts.

A recently published study on huntingtin's or *HTT* gene role in neurodevelopment in boys and girls (58) showed that in girls a longer CAG sequence in larger allele (still in normal range) correlated with thicker cortex and better cognition when in boys this impact was weaker being restricted mainly to lower putamen/cerebellum volume ratio in boys with higher CAG repeats number (58). This observation could suggest that also *HTT* gene mutation in women could exert stronger impact than in men.

The gender differences were identified in other neurodegenerative movement disorders caused by a dynamic mutation (CAG repeats expansion, similar to this causing HD, so called because number of CAG repeats may change during meiosis, or during mitosis) located in autosomal genes, namely in spinocerebellar ataxia (SCA) type 3 and 6 (13). In women, the progression of non-ataxia motor symptoms was faster than in men in those diseases. This effect in SCA type 3 was confirmed in a follow up study on the same European cohort (14). Moreover, faster progression in women with SCA 2 and 3 was reported in other studies (15, 16). This is consistent with findings in HD, suggesting that gender differences could be related to specific dynamic mutation mechanism, making it different than other monogenetic disorders.

Moreover, gender differences were described in various *CACNA1A* gene mutations, e.g., SCA 6 as an example of trinucleotides extension, episodic ataxia type 2 (EA2) in case of loss-of-function, and familial hemiplegic migraine-1 (FHM1) an example of gain-of-function missense mutation. In women, EA2 and FHM1 phenotypes were present when in men with the same mutation not (17). Interestingly also in *CACNA1S* gene mutation, which is another channel disorder, namely hypokalemic periodic paralysis, gender differences based on reduced penetrance in women and full in men were identified (18).

Gender differences in monogenetic autosomal neurological diseases do not always result in worsened progression or more severe clinical picture in one gender. This was confirmed by observation in Neurofibromatosis Type 1 (NF1). In a study conducted across girls and boys using “MOXO test,” which is being used for patients with ADHD, it was found that while the boys performed better than girls in attention and timing, they exhibited worse scores for impulsivity and hyperactivity. The observed difference does not comply with findings in general ADHD population, therefore a contribution of *NF1* gene mutation is likely (12). Gender differences in monogenetic neurologic disorders can be also race dependent as in the case of facioscapulohumeral muscular dystrophy type 1 (FSHD). The mutation in this disease similarly to this in HD is located in chromosome 4 and is related to nucleic acid length but in contrast refers to another place on the chromosome and results not from extension but from the contraction of D4Z4 repeats number. In a Korean study, it was identified that women are more seriously affected than men (1) when in studies based on European cohorts, men were more seriously affected (2–5). It was controlled for age and D4Z4 repeats number (1). In another neuromuscular monogenetic disease, namely Charcot–Marie–Tooth type 1A, women present a more severe phenotype and earlier onset age (6–9). It seems that gender differences, apart from those observed in SCA, in monogenetic neurological diseases do not reflect differences described in HD.

In monogenetic non-neurologic diseases, the more severe picture was described in men with autosomal dominant polycystic kidney disease (19, 20). In thalassemia major bone mass reduction was more prevalent and more severe in men. This finding was however accompanied by another one that women were more vulnerable for bones mass loss when hypogonadism co-existed, therefore, hormonal contribution seems to play an important role in observed differences (21). Hormonal factors partially explain more prevalent clinical manifestation of acute intermittent porphyria in women, in which the acute attacks occur rarely before puberty and its frequency and severity decline after menopause (10). Lower level of estrogens in postmenopausal women has been suggested to explain also more severe atherosclerosis in women affected by familial hypercholesterolemia (11).

Sex differences in common movement disorders were nicely summarized in a review published earlier this year (59). In movement's disorders, diseases' severity fluctuations related to menstrual cycle in women were reported in female patients with Parkinson's disease (60–62) and dystonia (63). It shed light on the effect of estrogens, indicating potential worsening in post-menopausal women (64). Future research in HD should, therefore, consider clinical differences between pre- and post-menopausal women included these on HRT to elucidate this effect in HD.

Currently, it is clear that there are gender differences in non-sex-linked genetic disorders. They are not well-understood; therefore, they ought to be investigated further, as they could shed light on disease mechanisms and pathogenesis. In diseases where gender differences were identified, the modeling, design, and interpretation of observational studies and clinical trials should be performed with respect to the gender of participants.

## Author Contributions

All authors listed have made a substantial, direct and intellectual contribution to the work, and approved it for publication.

## Conflict of Interest

The authors declare that the research was conducted in the absence of any commercial or financial relationships that could be construed as a potential conflict of interest.
